# Impact of Maternal Obesity on Liver Disease in the Offspring: A Comprehensive Transcriptomic Analysis and Confirmation of Results in a Murine Model

**DOI:** 10.3390/biomedicines10020294

**Published:** 2022-01-27

**Authors:** Beat Moeckli, Vaihere Delaune, Julien Prados, Matthieu Tihy, Andrea Peloso, Graziano Oldani, Thomas Delmi, Florence Slits, Quentin Gex, Laura Rubbia-Brandt, Nicolas Goossens, Stéphanie Lacotte, Christian Toso

**Affiliations:** 1 Hepatology and Transplantation Laboratory, Department of Surgery, Faculty of Medicine, Division of Visceral Surgery, University of Geneva, 1206 Geneva, Switzerland; beat.mockli@hcuge.ch (B.M.); vaihere.delaune@hcuge.ch (V.D.); andrea.peloso@hcuge.ch (A.P.); graziano.oldani@hcuge.ch (G.O.); thomas.delmi@hcuge.ch (T.D.); florence.slits@unige.ch (F.S.); quentin.gex@unige.ch (Q.G.); christian.toso@hcuge.ch (C.T.); 2 Department of Surgery, Division of Visceral Surgery, Geneva University Hospitals, 1205 Geneva, Switzerland; 3 Bioinformatics Support Platform, Services Communs de la Faculté, University of Geneva, 1206 Geneva, Switzerland; julien.prados@unige.ch; 4 Division of Clinical Pathology, Geneva University Hospitals, 1205 Geneva, Switzerland; matthieu.tihy@hcuge.ch (M.T.); laura.rubbia-brandt@hcuge.ch (L.R.-B.); 5 Division of Gastroenterology, Geneva University Hospitals, 1205 Geneva, Switzerland; nicolas.goossens@hcuge.ch

**Keywords:** maternal obesity, liver disease, whole-genome expression analysis, transcriptomics, non-alcoholic fatty liver disease, developmental origins of health and disease

## Abstract

The global obesity epidemic particularly affects women of reproductive age. Offspring of obese mothers suffer from an increased risk of liver disease but the molecular mechanisms involved remain unknown. We performed an integrative genomic analysis of datasets that investigated the impact of maternal obesity on the hepatic gene expression profile of the offspring in mice. Furthermore, we developed a murine model of maternal obesity and studied the development of liver disease and the gene expression profile of the top dysregulated genes by quantitative real-time polymerase chain reaction (qPCR). Our data are available for interactive exploration on our companion webpage. We identified five publicly available datasets relevant to our research question. Pathways involved in metabolism, the innate immune system, the clotting cascade, and the cell cycle were consistently dysregulated in the offspring of obese mothers. Concerning genes involved in the development of liver disease, *Egfr, Vegfb*, *Wnt2,* *Pparg* and six other genes were dysregulated in multiple independent datasets. In our own model, we observed a higher tendency towards the development of non-alcoholic liver disease (60 vs. 20%) and higher levels of alanine aminotransferase (41.0 vs. 12.5 IU/l, *p* = 0.008) in female offspring of obese mothers. Male offspring presented higher levels of liver fibrosis (2.4 vs. 0.6% relative surface area, *p* = 0.045). In a qPCR gene expression analysis of our own samples, we found *Fgf21, Pparg, Ppard*, and *Casp6* to be dysregulated by maternal obesity. Maternal obesity represents a looming threat to the liver health of future generations. Our comprehensive transcriptomic analysis will help to better understand the mechanisms of the development of liver disease in the offspring of obese mothers and can give rise to further explorations.

## 1. Introduction

We are living in a time of a global obesity epidemic with a global obesity rate that has doubled over the past 40 years and is predicted to reach 50% in the USA by 2030 [1,2]. Obesity is also associated with an elevated risk for several major chronic diseases such as type 2 diabetes, cardiovascular disease, some cancers, and chronic liver disease [3,4]. Women of reproductive age are particularly affected by obesity and close to one-third of children in the USA are born to obese mothers with a rising trend [5,6].

Besides increased perinatal complications, maternal obesity also leads to increased morbidity in the children of obese mothers [6,7,8]. In particular, maternal obesity is a risk factor for compromised cognitive development, metabolic syndrome, and even childhood cancers [9,10,11]. Additionally, murine studies have shown that pups born to obese mothers exhibit an increased risk of developing liver diseases such as steatosis and signs of hepatitis such as increased inflammatory markers and fibrosis [12,13]. A recent epidemiological study confirmed these results and demonstrated that the offspring of obese mothers had a threefold higher risk of developing non-alcoholic liver disease (NAFLD) in adulthood [14].

Several studies have explored the expression of selected genes, and a number of groups have performed whole-genome expression profiling [13,15,16,17,18,19,20,21,22] in the offspring of obese mothers. Study heterogeneity, the multifactorial etiology of the disease, and the comparatively small impact on gene expression by maternal obesity have not allowed clear identification of the molecular pathways involved in the development of chronic liver disease. The aim of this study is to identify the major pathways and genes associated with the development of liver disease in the offspring of obese mothers.

Due to the heterogeneity of the available data, we chose to perform an integrative genomic analysis of all available datasets studying the transcriptional impact of maternal obesity. This allowed us to identify genes and pathways that were dysregulated most consistently among the different studies. In the next step, we developed our own model of maternal obesity and characterized the offspring. We then used the information that we obtained from the integrative genomic analysis to study the most consistently dysregulated genes in our own samples. The raw results of our analysis are publicly available for different analyses by other groups combined with a web-based tool for the analysis of specific genes (http://www.genebrowser.unige.ch/maternalobesity/, accessed on 29 November 2021).

## 2. Materials and Methods

### 2.1. Database Search

We performed a search of the Gene Expression Omnibus (GEO) [23] from the National Center for Biotechnology Information for gene expression datasets using the following search term ((“obesity”[MeSH Terms] OR obese[All Fields]) OR fat[All Fields] OR western[All Fields]) AND (F1[All Fields] OR offspring[All Fields]) AND ((“liver”[MeSH Terms] OR liver[All Fields]) OR hepatic[All Fields]). Only mouse studies were included in our analysis. Additionally, the Center for Information Biology Gene Expression database (CIBEX) [24] and ArrayExpress [25] were searched with similar search terms but no additional hits were retrieved (Figure S1).

### 2.2. Qualtitative Literature Search to Identify Pathways and Genes Relevant for the Progression of Liver Disease

We performed a qualitative literature search to identify genes and pathways that have been identified previously as relevant for the development or the progression of non-alcoholic fatty liver disease (NAFLD) or non-alcoholic steatohepatitis (NASH). Additionally, we included genes that were identified by other groups to play a role in the development of liver disease in the offspring of obese mothers. A full list of pathways and genes with references is available under Supplementary Materials (Tables S1 and S2).

### 2.3. Gene Expression Analysis

All transcriptomic data analyses were performed in R (version 4.0.4, R Foundation for Statistical Computing, Vienna, Austria) [26]. The raw data of the identified microarray datasets were downloaded with the get GEO function of the GEOquery package (version 2.58.0, Bioconductor, Buffalo NY, USA) [27]. Raw data for the RNA sequencing dataset (GSE134976) were downloaded directly from GEO as a text file containing RPKM (Reads Per Kilobase of transcript per Million mapped reads) for each gene analyzed. All datasets were already normalized. For the differential gene expression analysis, we used the limma package (version 3.46.0, Bioconductor, Buffalo NY, USA) with the lmFit and eBayes functions [28]. This analysis yields an estimate of log fold change between conditions, a significance *p*-value, and a confidence interval for the log fold change.

### 2.4. Pathway Analysis

All genes found in at least one of the datasets were annotated with the Reactome pathways they are associated with [29,30]. Out of the 8488 annotated genes, we identified the 250 most up- and 250 most down-regulated in each study. This was achieved by ranking the genes according to their log2 fold-change, excluding those with a *p*-value above 0.05. Then, a hypergeometric test was conducted to identify enriched pathways in each of the selected gene sets.

### 2.5. Webpage Design

The companion webpage to this publication was built in R (version 4.0.4) with the shiny package [31]. The entire code for this webpage is available under the following repository: www.github.com/moecklib/IGA_maternalobesity/ (accessed on 29 November 2021).

### 2.6. Animal Experimentation

Animal experimentation was carried out under the terms of an experimental protocol approved by the ethical committee of the University of Geneva and the Geneva veterinary authorities (GE31-18). The project was originally approved on the 01.09.2017 by the veterinary authorities. All mice were housed in the animal facility of the University of Geneva under 12/12 h light/dark cycles with free access to food and water. Dams (C57BL/6N) were purchased from Charles River Laboratories (Ecully, France). They were then randomly assigned to either a control diet (ND: 17% kcal fat, 61% kcal carbohydrate, 22% kcal protein; Envigo TD.120455) or an energy-rich Western-style high-fat diet (HFD: 45% kcal fat, 41% kcal carbohydrate, 15% kcal protein; Envigo TD.08811).

After 12 weeks on their respective diets, the female mice were mated with males on a control diet. Throughout pregnancy and lactation, dams received the same diet as before mating (ND or HFD). Offspring were weaned at 21 days of age to different cages depending on their sex and the maternal diet. All offspring received a control diet (ND). A blood draw by puncture of the tail vein and an oral glucose tolerance test were performed at 24 weeks of age and mice were sacrificed by exsanguination under isoflurane anesthesia at 40 weeks of age. Blood was collected by puncture of the inferior vena cava and livers were dissected and weighed. Liver samples were snap-frozen and stored at −80 °C until further use. The rest of the liver was placed in buffered 4% formaldehyde solution for later histological analysis. Both female and male offspring were included in the analysis forming the following groups: Female offspring of HFD mothers (F_HFD), male offspring of HFD mothers (M_HFD), female offspring of ND mothers (F_ND), and male offspring of ND mothers (M_ND).

### 2.7. Oral Glucose Tolerance Test

Mice were fasted for 6 h and received 2 g/kg body weight of a 100 mg/mL glucose solution by oral gavage. Blood was collected before, and at 5, 10, 15, 30, 60, 90, and 120 min after the administration of glucose to measure glucose levels using an Accu-Check glucometer (Roche Diabetes Care, Rotkreuz, Switzerland).

### 2.8. Plasma Analysis

Serum alanine aminotransferase (ALT) was analyzed using the veterinary chemistry analyzer ReflovetTM Plus (Roche Diagnostic, Rotkreuz, Switzerland).

### 2.9. Histology

Offspring livers at 40 weeks of age were fixed in buffered 4% formaldehyde solution and paraffin embedded before sectioning. All sections were stained with hematoxylin and eosin and Masson’s trichrome to assess steatosis, inflammation, and fibrosis. A threshold of 5% of hepatocytes with macrovesicular steatosis was required for the diagnosis of NAFLD. NASH was defined as a global pattern of histological damage based on the presence of > 5% macrovesicular steatosis, inflammation, and ballooning of hepatocytes according to the Bedossa scoring system [32].

#### 2.9.1. Assessment and Quantification of Steatosis

Quantification of steatosis was performed with a python script based on HE staining and adapted from scikit-image [33]. Sagittal sections of a whole liver lobe were included in the analysis. Whole slide images were scanned using a PANNORAMIC 250 Flash scanner (3DHISTECH Kft., Budapest, Hungary). The nucleus and vacuoles of steatosis were automatically detected and validated visually. The relative area of steatosis was calculated as the area of vacuoles normalized by the total area of the whole slide.

#### 2.9.2. Assessment and Quantification of Fibrosis

Fibrosis quantification was performed with the QuPath software package (version 0.3.2, QuPath, Edinburgh, UK) [34]. Two sagittal sections of an entire liver lobe per animal were included in the analysis. Whole slide images with Masson’s Trichrome stain were scanned with the Zeiss Axioscan.Z1 Microscope Slide Scanner (Carl Zeiss AG, Jena, Germany). Parts of the slide containing tissue were detected in QuPath using the createAnntationFromPixelClassifier function. The thereby created annotations were manually assessed and cleaned to only include liver tissue without large vessels or purely fibrotic tissue. Next, parts of the whole slide image containing fibrotic tissue, stained in blue, were quantified with a manually trained tissue thresholder. Once more, the thereby detected fibrotic tissue was assessed blindly to only include fibrotic tissue. The relative surface of fibrosis was calculated as the surface of the whole slide image classified as fibrosis divided by the total surface of the whole slide image.

### 2.10. Gene Expression Analysis

After total RNA extraction of liver tissue (ReliaPrepTM RNA Tissue, Promega, Madison WI, USA), cDNA was synthesized by extending a mix of random primers with the High-Capacity cDNA Reverse Transcription Kit in the presence of an RNAase inhibitor (Applied Biosystems). The relative quantity of each transcript was normalized to the expression of *Eef1*, *Hprt*, and *Gapdh*. SYBRGreen reagent was used for real-time quantitative polymerase chain reaction (qPCR) on the ABI Prism 7000 sequence detection system (Applied Biosystems) according to the manufacturer’s instructions. Primer sequences are provided in Supplementary Table S3.

### 2.11. Statistical Analysis

No data were excluded. Statistical significance between two groups was calculated using the Mann–Whitney *U*-test. All statistical analyses were performed using R (version 4.0.4, R Foundation for Statistical Computing, Vienna, Austria) [26]. Figures display the median ± interquartile range. A *p*-value < 0.05 was considered statistically significant.

## 3. Results

### 3.1. Characteristics of the Included Datasets

Five gene expression datasets deposited in the Gene Expression Omnibus repository with two different analyzed conditions were included for further analysis [17,18,19,20]. All datasets compared the whole-genome expression profile in liver tissue of the offspring of obese mothers compared to the offspring of lean mothers (Table 1). The selected studies included samples from 124 animals and were published between 2013 and 2019. All studies included two different comparisons, differentiating between diet, sex, or age of the offspring (Table 1). All studies published their results in peer-reviewed journals, except for one study (GSE134976) which has not yet been published. One dataset (GSE46359) studied gene expression in pups of two weeks of age and all other datasets studied gene expression in adult mice. One dataset studied the gene expression in female offspring (GSE46359_F), all other datasets used male offspring.

### 3.2. Pathway Analysis in the Offspring of Obese Mothers

**Figure 1:**
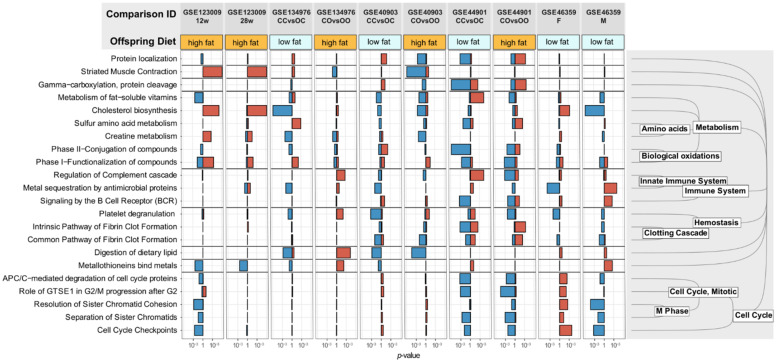
Dysregulated pathways in offspring of obese mothers. Display pathways that were differentially regulated in at least three different GEOSET with a significance threshold of *p* < 0.01. The Reactome Pathway Database (www.reactome.org (accessed on 29 November 2021)) was used for annotation of the pathways. A blue bar indicates a downregulation of the corresponding pathway, a red bar an upregulation. The legend to the right of the figure depicts the hierarchical clustering of the dysregulated pathways according to Reactome. The comparison GSE46359_F studied female offspring, all other comparisons studied male offspring.

In each individual dataset, we identified the 500 most dysregulated genes (250 up- and 250 down-regulated) and performed pathway enrichment analysis on each of them. Pathways that were differentially regulated in at least three different comparisons with a significance threshold of *p* < 0.01 were considered dysregulated pathways (Figure 1).

Out of 1692 pathways in Reactome, the Gene Set Enrichment Analysis revealed 22 dysregulated pathways in offspring of obese mothers. As expected, a large part (10 out of 22 pathways) concerned metabolic processes. More interestingly, the complement cascade and the metal sequestration of antimicrobial proteins pathways were dysregulated in three of the four datasets. Both pathways are involved in the innate immune system. Furthermore, in a majority of the datasets (6 out of 10 datasets), at least one pathway involved in the clotting cascade was either up- or downregulated. Finally, five dysregulated pathways involved the cell cycle and cell division (cell cycle checkpoints, cell cycle protein degradation, and sister chromatid separation). Interestingly, genes of the cell cycle pathways were regulated in opposite directions in female and male offspring of obese mothers.

### 3.3. Analysis of Top Dysregulated Genes in the Offspring of Obese Mothers

**Figure 2:**
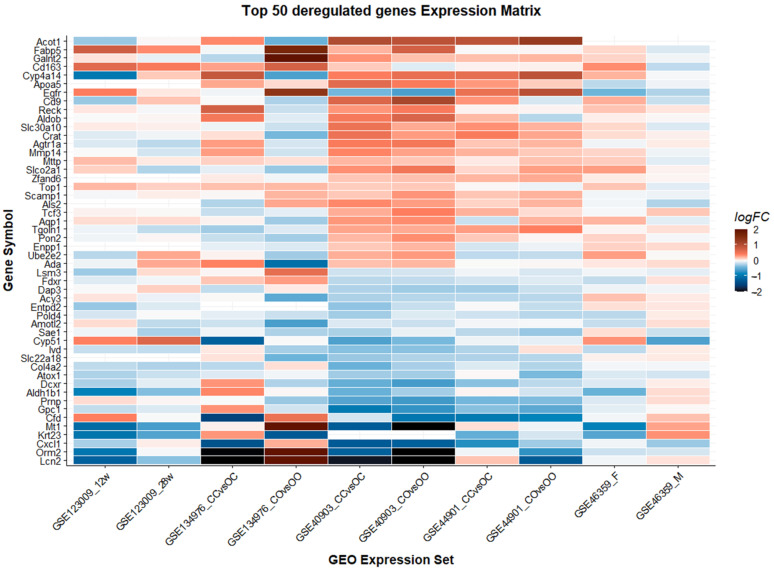
Display of the 50 most dysregulated genes (> 0.1 LogFC and *p* < 0.05 in > 3 datasets). Genes are ranked according to their combined logarithmic fold change from highest to lowest. The comparison GSE46359_F studied female offspring, all other comparisons studied male offspring.

We defined consistently differentially regulated genes as the following: Genes that are differentially expressed between offspring of obese and lean mothers with a significance level of *p* < 0.05 and a log2 fold change (logFC) > 0.1 in at least three different conditions, or two independent experiences. We identified 306 genes meeting these criteria. Of these 306 genes, 171 are annotated in the Reactome database [29,30]. A search flow visualizing the selection process is available in the Supplementary Materials (Figure S2). We used annotation in the Reactome database as a proxy for clinical and biological significance and used only annotated genes for further analysis. We ordered the obtained gene list according to the number of experiments in which the genes are differentially up- or downregulated (logFC > 0.1, *p* < 0.05), the median logFC, and the classical Fisher’s *p*-value combination method [35]. The 50 most dysregulated genes are displayed in Figure 2 and the whole table is available as a Supplementary Table (Table S4).

Five genes were differentially expressed in at least five different conditions (*Acy3*, *Galnt2*, *Agtr1a*, *Sae1*, *Mttp*). Not surprisingly, most of these genes are involved in metabolic processes except for *Agtr1*—a gene encoding angiotensin II receptor 1. This is an important effector in blood pressure control and has been associated with a deleterious effect on the progression of NAFLD [36]. *Agtr1* is upregulated in five out of ten datasets. The gene with the highest median fold change, *Lcn2*, encodes a protein that is involved in the defense of the host against invading bacteria and is induced by toll-like receptors [37]. Other genes that are differentially regulated with a high median fold change include *Orm2*, an acute phase reactant, *Cfd* involved in the complement cascade, and *Egfr* a tyrosine kinase, which is upregulated in a number of different cancer types [38,39,40].

To identify genes that could potentially be responsible for the increased risk for chronic liver disease in the offspring of obese mothers, we selected genes that are members of previously identified molecular pathways involved in the development of NAFLD or NASH (Table S1). Our list of differentially regulated genes involved in these pathways includes ten genes. Their function, associated pathways, and gene expression characteristics are summarized in Table 2.

### 3.4. Validation of Previously Identified Molecular Targets

**Figure 3:**
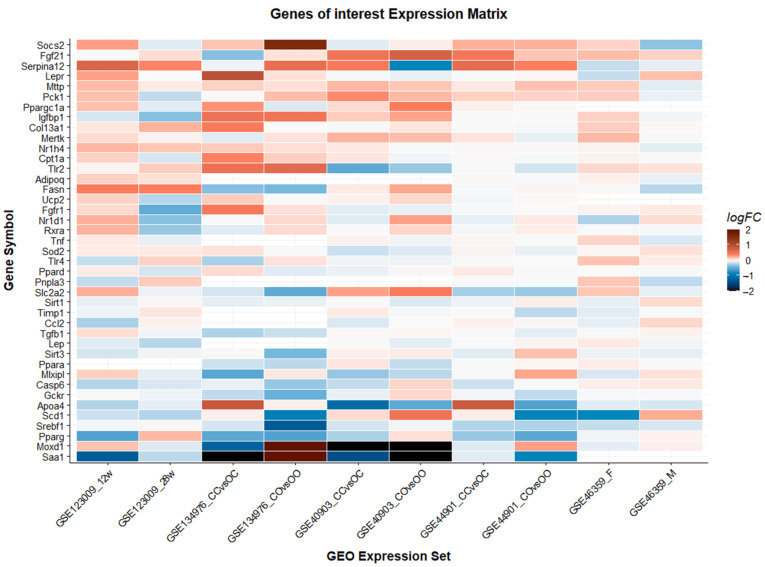
Validation of previously identified genes. Genes are ranked according to their combined logarithmic fold change from highest to lowest. The comparison GSE46359_F studied female offspring, all other comparisons studied male offspring.

In addition to identifying new genes and pathways responsible for the development of liver disease in the offspring of obese mothers, we also aimed to validate previously proposed genes. We performed a literature review to identify genes that were dysregulated in the offspring of obese mothers in individual studies or are associated with the development or progression of NAFLD. We obtained a list of 44 genes that we validated against the ten individual comparisons in this study (Figure 3, Table S2).

Thirty-four of these “genes-of-interest” were annotated in the Reactome database and five out of the 34 annotated genes were differentially expressed in at least three different datasets (*Mttp, Pparg, Slc2a2, Srebf1, Mertk*). We applied the same criteria mentioned above under 3.3 to select genes implicated in relevant pathways for the development or progression of NAFLD (Table S5). A selection of these genes with a Fisher’s *p*-value of <0.01 is included in Table 3. For further exploration of individual genes, please use our interactive companion webpage: www.genebrowser.unige.ch/maternalobesity/ (accessed on 29 November 2021).

### 3.5. Companion Webpage

To aid future research in this important topic, we decided to publish a companion webpage available under www.genebrowser.unige.ch/maternalobesity/ (accessed on 29 November 2021), including the data of the integrative genomic analysis. The webpage has several features; it allows to visually explore the expression of a given gene over a selection of comparisons and provides the level of expression of the selected gene in percentile for each GEOSET. The “Heatmap” tab allows selecting a list of genes based on varying selection criteria (minimal logFC, maximal *p*-value, and the number of comparisons fulfilling the conditions). The “Volcano Plot” tab visualizes the logFC and *p*-value of a selected gene in regard to all other genes included in the study. Finally, the “GEOSET description” tab provides a tabularized description of each included study.

The companion webpage adds value to this manuscript in several regards. It allows researchers to explore their respective gene of interest in only the conditions they are interested in. Additionally, it provides an overview of the expression level of a gene in each study. This is especially important since we did not apply a minimal expression threshold for a gene to be included in our integrative genomic analysis. Lastly, researchers can perform their own data exploration and identify new genes that may play a role in the transmission of disease only under certain circumstances.

### 3.6. Maternal Obesity Causes Liver Disease

**Figure 4:**
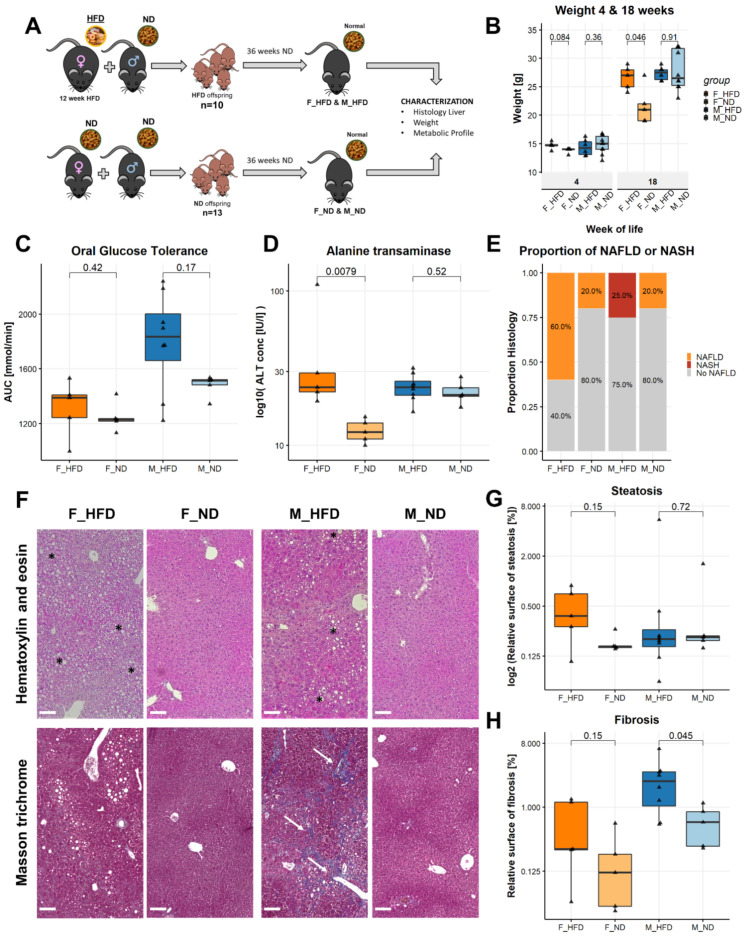
Maternal obesity causes liver disease in offspring. (**A**) Experimental setup, mothers received high-fat diet for 12 weeks before mating, offspring were sacrificed at 40 weeks of age. (**B**) Weight of the offspring at the time of weaning and at 18 weeks of age. (**C**) Oral glucose tolerance test. The area under the curve (mmol/min) measured over 120 min is represented. (**D**) Alanine transaminase levels in the serum measured at 40 weeks. (**E**) Proportion of animals with non-alcoholic fatty liver disease (NAFLD) or non-alcoholic steatohepatitis (NASH) according to the criteria by Bedossa et al. (**F**) Representative histological images. The upper row represents hematoxylin and eosin stained tissue slides and the lower row masson trichrome stained slides. The black asterisks mark areas with steatosis and the white arrows areas with fibrosis. (**G**) Quantification of steatosis defined as tissue surface covered by lipid vacuoles divided by total surface. (**H**) Quantification of fibrosis defined as tissue surface covered by fibrosis divided by total surface. Statistical analysis: Wilcoxon signed-rank test (**B**,**C**,**D**,**E**,**G**,**H**). Median with interquartile ranges. F_HFD: Female offspring born to obese mothers *n* = 5, F_ND: Female offspring born to lean mothers *n* = 5, M_HFD male offspring born to obese mothers *n* = 8, M_ND male offspring born to lean mothers *n* = 5. Scale bar Figure 4F: 100µm.

To study the effect of maternal diet on the development of liver disease in the offspring, we assigned female C57BL/6N mice to either a high-fat diet (HFD) or a normal diet (ND) for 12 weeks before mating them with lean males. After birth and during lactation, the offspring remained with the mother and their respective diet (HFD or ND) for four weeks. After weaning, all offspring received a normal control diet (ND) for 36 weeks (Figure 4A). At weaning, we did not observe significant weight differences in female or male offspring. However, female offspring of obese mothers gained significantly more weight during the course of their life (Figure 4B). We observed a trend towards decreased glucose tolerance in males at 24 weeks; however, this did not reach statistical significance (1835 vs. 1515 mmol/min, *p* = 0.17) (Figure 4C). To assess for liver disease, we measured serum alanine transaminase in the serum at 24 weeks of age. Alanine transaminase levels were significantly elevated in female offspring of obese mothers (23.8 vs. 12.2, *p* = 0.0079) (Figure 4D). A higher proportion of female offspring of obese mothers developed NAFLD at 40 weeks of age (60 vs. 20%) and two out of 13 male offspring of obese mothers developed NASH versus none in the other groups (15.4 vs. 0%) (Figure 4E). A representative histological section of a male with NASH is shown in Figure 4F. In order to quantify the amount of steatosis, we measured the vacuole surface over the total tissue surface. Female offspring of obese mothers exhibited a trend towards a higher percentage of tissue surface occupied by vacuoles and therefore a higher amount of steatosis (0.38 vs. 0.16% relative vacuole surface, *p* = 0.15). In male offspring, we did not observe a difference in steatosis. (Figure 4F,G). Regarding fibrosis, male offspring of obese mothers had a significantly higher amount of fibrosis than male offspring of lean mothers (2.37 vs. 0.61% relative fibrosis surface, *p* = 0.045) (Figure 4F,H).

### 3.7. Maternal Obesity Dysregulates Genes of the Peroxisome Proliferator Activated Receptor and Caspase Pathways in Offspring

**Figure 5:**
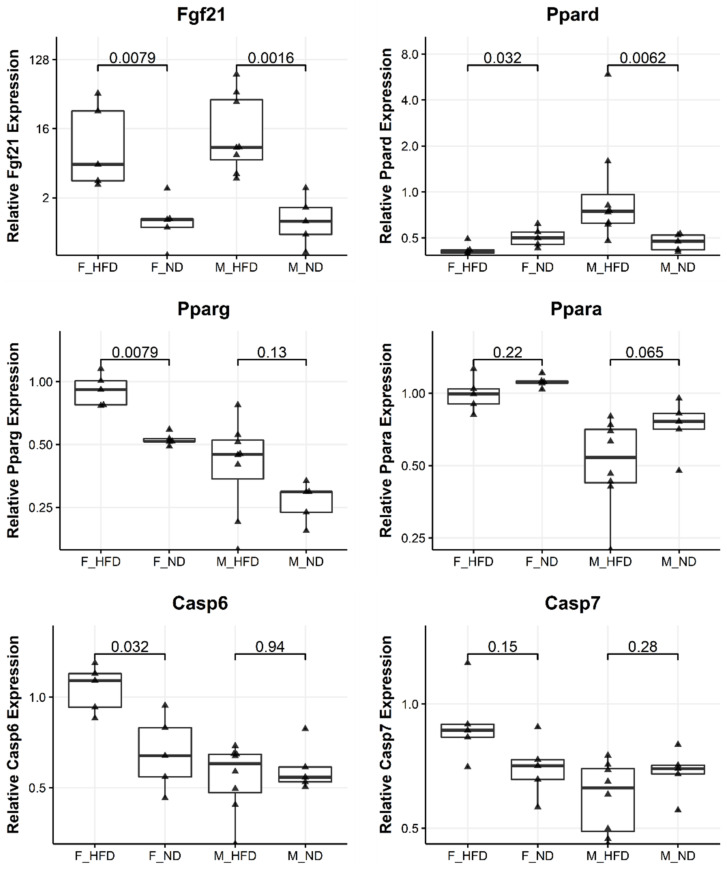
Maternal obesity dysregulates genes of the peroxisome proliferator-activated receptor and caspase pathways in offspring. Quantitative real-time polymerase chain reaction gene expression results comparing the gene expression between offspring of obese and lean mothers. F_HFD: Female offspring born to obese mothers *n* = 5, F_ND: Female offspring born to lean mothers *n* = 5, M_HFD male offspring born to obese mothers *n* = 8, M_ND male offspring born to lean mothers *n* = 5. Statistical analysis: Wilcoxon signed-rank test. Results are expressed as Median with interquartile ranges.

In order to link the development of liver disease in the offspring of obese mothers with underlying molecular changes, we performed an expression analysis by qPCR in our own model of maternal obesity. We chose the top dysregulated genes, implicated in the development and progression of liver disease identified by the integrative genomic analysis (Table 2). Furthermore, we included the genes from the qualitative literature search that were differentially expressed in at least three different datasets or associated with the development of NAFLD (Table S4). This yielded a total number of 23 genes that we included in our gene expression analysis (Figure S3). Of these, five were significantly dysregulated (*p* < 0.05) in the liver of either male or female offspring of obese mothers (*Fgf21, Pparg, Ppard, Casp6, Srebf1*). A further three genes showed differences between the offspring of obese and non-obese mothers with a *p* < 0.01 (*Ppara, Tlr2, Vim*). Four out of five differentially expressed genes belong to either the peroxisome proliferator-activated receptor or the caspase pathways [41,42,43] (Figure 5). Caspase genes are involved in apoptosis; however, a mere change in expression does not allow drawing any conclusions on the presence or absence of apoptosis.

The gene with the highest differential expression in our own samples was fibroblast growth factor 21, a major hepatokine involved in glucose homeostasis, insulin sensitivity, and lipid metabolism [44]. This gene was upregulated five times in female offspring and nine times in male offspring of obese mothers (Figure 5). The regulation of *Fgf21* is closely intertwined with the peroxisome proliferator-activated receptor pathway. Indeed, we observed an increased expression of *Pparg*, a gene known to be induced by *Fgf21* [45]. However, *Ppara*, which regulates the expression of *Fgf21* was not significantly upregulated in our samples [46].

As seen in the integrative genomic analysis, we observed marked differences in gene expression changes between males and females in response to maternal obesity. For instance, expression of *Casp6* is upregulated in female offspring of obese mothers but unchanged in male offspring, *Ppard* is downregulated in female offspring of obese mothers and upregulated in male offspring of obese mothers, and *Fgf21* is upregulated in both female and male offspring of obese mothers.

## 4. Discussion

The rising global obesity rate is a major public health concern for future generations. Obesity of the mother places the offspring at risk for developing NAFLD [14]. However, the mechanisms involved remain unclear. In this study, we performed a comprehensive analysis of all available datasets studying the effect of maternal obesity on the gene expression in offspring and identified major pathways and genes involved in the development of liver disease. Furthermore, we demonstrate that the offspring of obese mothers have a higher risk of developing liver disease, even when fed a normal diet. The results of our integrative genomic analysis can be explored on our companion webpage (www.genebrowser.unige.ch/maternalobesity/ (accessed on 29 November 2021)).

Genes involved in lipid metabolism were amongst the genes most consistently differentially regulated in the different datasets. For example, *Mttp*, a gene that encodes for microsomal triglyceride transfer protein, is upregulated in five different conditions out of ten [47]. This protein plays a central role in the lipoprotein assembly and is the target of lomitapide, an approved lipid-lowering agent for the treatment of familial hypercholesterolemia [48]. This could be one possible explanation for the increased risk for dyslipidemia in children of obese mothers [49]. Another gene consistently upregulated in five out of eight comparisons studying adult offspring is *Agtr1* coding for angiotensin receptor 1. The renin–angiotensin system has been implicated repeatedly in the progression of chronic liver disease [50,51,52,53]. Angiotensin II binds to the angiotensin II type I receptor and leads to the activation of hepatic stellate cells and in turn deposition of collagen and other components of the extracellular matrix [54]. This potentially explains the increased rate of fibrosis that we observed in male offspring of obese mothers.

Moreover, we found a number of pro-oncogenic genes to be consistently upregulated by maternal obesity in multiple independent experiments. Namely, *Egfr, Vegfb, Wnt2*, and *Wnt5b*, are all genes that play a role in the development or progression of hepatocellular carcinoma [55,56,57]. Epidemiological studies have linked maternal obesity to childhood cancers and colorectal carcinoma in the offspring of obese mothers [11,58]. A recent murine study showed that the offspring of obese mothers are at a higher risk of developing hepatocellular carcinoma [59]. The authors linked this transgenerational effect to the gradual downregulation of genes involved in the acylation of fatty acids and the normalization of the mitochondrial acetaldehyde redox level. An effect of maternal obesity on hepatocellular carcinoma in humans has not yet been established. Future large-scale epidemiological studies are needed to answer this question. Our results provide insight into potential molecular mechanisms contributing to the increased susceptibility of obese mothers’ offspring to develop cancers.

One of the goals of this study was to validate target genes identified by the integrative genomic analysis in our own model. For validation, we chose significantly dysregulated genes that also belong to a pathway involved in chronic liver disease. The most dysregulated gene that we validated in our own samples was *Fgf21*. This major hepatokine has a myriad of different functions. Among others it regulates glucose homeostasis and hepatic lipid metabolism but also seems to play a role in chronic liver disease. Patients with NAFLD have higher serum levels of FGF21 [60]. Several FGF21 analogs are in clinical development for the treatment of NAFLD and have shown success in reducing hepatic fat content [61,62]. The second most expressed gene that we validated in our samples is *Pparg*, a gene that belongs to the same pathways as *Fgf21* [45,63]. It remains to be clarified if the upregulation of *Fgf21* in the offspring of obese mothers is a mere consequence of liver disease or plays an active role in its development.

We observed marked differences between males and females in the response to maternal obesity. For example, cell cycle, innate immune system, and cholesterol pathways were oppositely regulated in the GSE46359 dataset, which was the only study in our analysis that included both males and females [19]. In our own model, female offspring of obese mothers exhibited an increased risk for NAFLD and male offspring of obese mothers for fibrosis. One possible explanation is that male offspring of obese mothers are equally likely to develop steatosis but are more vulnerable to developing NASH once there is steatosis. On the other hand, female offspring of obese mothers seem more likely to develop steatosis but are protected from developing NASH. This emphasizes the fact that sex plays an important role in the development of liver disease. Furthermore, certain genes (*Ppard, Pparg*, and *Casp6*) were expressed differentially between female and male offspring. These results are in line with the findings of other groups, which have shown different effects of maternal obesity on male and female offspring in regard to glucose homeostasis, lipid metabolism, and hepatic steatosis [15,64,65]. Our results further highlight the importance of studying the effect of maternal obesity on both males and female offspring. A shortcoming of the current literature is that it has mainly focused on male offspring.

It is noteworthy that in our model, the offspring of obese mothers developed liver disease without external induction through, for example, an obesogenic diet. All our offspring received a control non-high-fat diet. This supplements previously published studies, which challenged offspring with an obesogenic diet [13,16,66]. For the first time, we confirmed that the offspring of obese mothers have a higher risk for developing fibrosis on a control diet. Only a subset of offspring of obese mothers developed steatosis and an even smaller proportion developed fibrosis and NASH. This is likely representative of what happens in clinical reality [14]. It is, therefore, crucial to identify and follow the individuals at risk as early as possible. Molecular signatures have been proposed to identify patients at increased risk for the progression of chronic liver disease [67,68,69]. Similarly, the results of our integrative genomic analysis could represent a first step towards identifying key genes dysregulated in young children of obese mothers who are at risk of developing liver disease later in life.

Primarily two mechanisms have been suggested by which maternal obesity could affect the gene expression in the offspring. One hypothesis is that epigenetic changes in the offspring genome occur in utero and during lactation [22,70,71]. The other possibility is the transmission of an altered microbiome from the obese mother to the offspring during the perinatal period [64]. The altered gut microbiome in obese patients can lead to a progression of liver disease and is itself transmitted to the offspring [21,72]. We observed an upregulation of genes involved in the innate immune response in the livers of the offspring of obese mothers (Figure 1): This could be the effect of increased stimulation of the hepatic immune system by bacterial endotoxins from the enterohepatic circulation and therefore supports the second hypothesis [73,74].

Our study has several limitations. The included studies in our integrative genomic analysis are heterogeneous in regard to the age and diet of the offspring and the mother. We partially addressed this shortcoming by offering the possibility for a differentiated search on the companion webpage of this publication. Furthermore, we relied on the Reactome pathway database for the annotation and selection of genes. It is possible that unannotated genes play an important role in the transmission of liver disease from mother to offspring but were not included in our analysis. We provide a complete list of all 45,944 genes analyzed in this study in Table S6. Additionally, the increased expression of caspase 6 in female offspring of obese mothers does not necessarily correlate with an increased caspase activity. Finally, we narrowed the confirmation of gene targets in our own samples mainly to genes previously associated with the progression of NAFLD. It could be of interest to extend this confirmation to genes involved in other pathways such as *Mttp1* or *Agtr1*.

The current obesity epidemic represents a looming threat to the liver health of future generations. Our comprehensive transcriptomic analysis discovered new pathways and molecular targets. This will help to better understand the mechanisms of the development of liver disease in the offspring of obese mothers.

## Figures and Tables

**Table 1:** Characteristics of the included datasets on *Mus musculus* liver tissue.

Comparison ID	GEO Accession Number	Publication	Year	Group	Samples/Group	Sex	Age	Technique
GSE123009_12w	GSE123009	Rouschop et al., J Lipid Res, 2019 [18]	2019	CO vs. OO	7	M	12 w	MicroArray
GSE123009_28w	GSE123009	Rouschop et al., J Lipid Res, 2019 [18]	2019	CO vs. OO	9	M	28 w	MicroArray
GSE134976_COvsOO	GSE134976	Pantaleao et al., unpublished	2019	CO vs. OO	3	N/A	12 w	RNA-seq
GSE134976_CCvsOC	GSE134976	Pantaleao et al., unpublished	2019	CO vs. OO	3	N/A	12 w	RNA-seq
GSE40903_COvsOO	GSE40903	Cannon et al., PLoS One, 2014 [17]	2014	CO vs. OO	9	M	9 w	MicroArray
GSE40903_CCvsOC	GSE40903	Cannon et al., PLoS One, 2014 [17]	2014	CC vs. OC	9	M	9 w	MicroArray
GSE44901_CCvsOC	GSE44901	Pruis et al., Acta Physiol, 2014 [20]	2014	CO vs. OO	5	M	29 w	MicroArray
GSE44901_OCvsOO	GSE44901	Pruis et al., Acta Physiol, 2014 [20]	2014	CC vs. OC	5	M	29 w	MicroArray
GSE46359_F	GSE46359	Mischke et al., PLoS One, 2013 [19]	2013	CC vs. OC	6	F	2 w	MicroArray
GSE46359_M	GSE46359	Mischke et al., PLoS One, 2013 [19]	2013	CC vs. OC	6	M	2 w	MicroArray

Abbreviations: GSE: Gene set enrichment; CC: Mother and offspring fed control diet; CO: Mother fed control diet and offspring fed obesogenic diet; OC: Mother fed obesogenic diet and offspring control diet; OO: Mother and offspring fed obesogenic diet; M: Male; F: Female; RNA-seq: RNA sequencing.

**Table 2:** Differentially regulated genes involved in the development and progression of chronic liver disease.

Gene	Gene Name	Function	Main Associated Pathways	Nb Comparison Up	Nb Comparison Down	Median logFC	Fisher’s *p*-Value
*Egfr*	Epidermal growth factor receptor	Receptor tyrosine kinase with a multitude of downstream functions. Implicated in a wide variety of cancers.	ERRB signaling; MAPK-Erk; PI3K/AKT	4	2	0.418	2.1 × 10^−8^
*Pparg*	Peroxisome proliferator-activated receptor gamma	Nuclear receptor involved in critical metabolism regulations of various cell types, notably adipocyte differentiation.	MAPK-Erk	0	3	0.268	1.8 × 10^−3^
*Vegfb*	Vascular endothelial growth factor B	Encodes VEGF-B protein, which has anti-apoptotic effects, including neuroprotection. Promotes blood vessel survival.	MAPK-Erk; PI3K/AKT	3	0	0.263	3.0 × 10^−9^
*Wnt2*	Wingless-type family, member 2	Encodes proteins involved in Wnt signaling pathway, oncogenesis, embryonic patterning, and cell fate commitment.	Wnt signaling pathway	3	0	0.249	2.7 × 10^−3^
*Nrp1*	Neuropilin 1	Tyrosine kinase coreceptor for VEGFs and semaphorins. Participates in angiogenesis and several developmental pathways.	VEGF signaling pathway	3	0	0.219	8.7 × 10^−5^
*Vim*	Vimentin	Protein encoding gene for mesenchymal class-III intermediate filaments.	Apoptosis	0	3	0.146	7.1 × 10^−3^
*Tle1*	Transducin-like enhancer of split 1	Transcriptional repressor protein essential in embryogenesis, hematopoiesis, and epithelial differentiation. Expressed in certain tumors.	Wnt signaling pathway; NF-kappa-B	3	0	0.128	1.2 × 10^−2^
*Casp7*	Caspase-7	Apoptosis-related cysteinyl aspartate proteinase. Protein coding gene for cell death execution via activation cascades.	Apoptosis	0	3	0.113	9.5 × 10^−7^
*Wnt5b*	Wingless-type family, member 5b	Encodes secreted signaling protein that play a role in developmental signaling, proliferation, migration, and tumorigenesis.	Wnt signaling pathway; PCP/CE pathway	0	3	0.109	4.2 × 10^−9^
*Daam1*	Dishevelled-associated activator of morphogenesis 1	Intracellular protein involved in actin cytoskeleton functions.	Wnt signaling pathway; Rho GTPases signaling	0	3	0.101	2.3 × 10^−4^

Abbreviations: Nb comparison up: Number of gene enrichment sets in which the gene is upregulated; Nb comparison down: Number of gene enrichment sets in which the gene is downregulated; LogFC: log2 fold change.

**Table 3:** Expression of previously identified molecular targets involved in chronic liver disease with a Fisher’s *p*-value of <0.01.

Gene	Gene Name	Function	Main Associated Pathways	Nb Comparison Up	Nb Comparison Down	Median logFC	Fisher’s *p*-Value
*Pparg*	Peroxisome proliferator-activated receptor gamma	Nuclear receptor involved in critical metabolism regulations of various cell types, notably adipocyte differentiation.	MAPK-Erk	0	3	0.268	1.8 × 10^−3^
*Tlr2*	Toll-like receptor 2	Membrane surface receptor essential for pathogen recognition and innate immune response activation.	NF-kappa-B; MAPK-Erk	0	2	0.135	3.3 × 10^−2^
*Fgf21*	Fibroblast growth factor 21	Hepatokine involved in mitogenic activities. Major regulator of energy homeostasis.	MAPK-Erk; Insulin signaling	2	0	0.131	0.079
*Fgfr1*	Fibroblast growth factor receptor 1	Receptor tyrosine kinase that plays a fundamental role in embryogenesis and cell development.	MAPK-Erk; PI3K/AKT	0	1	0.064	0.092

Abbreviations: Nb comparison up: Number of gene enrichment sets in which the gene is upregulated; Nb comparison down: Number of gene enrichment sets in which the gene is downregulated; LogFC: log2 fold change.

## Data Availability

The datasets analyzed during the current study are available in the Gene Expression Omnibus repository https://www.ncbi.nlm.nih.gov/geo/ (accessed on 29 November 2021). All code for data cleaning and analysis associated with this manuscript is available under www.github.com/moecklib/IGA_maternalobesity (accessed on 29 November 2021). Any updates during the current submission process will be added to this repository.

## Supplementary Materials

The following are available online at https://www.mdpi.com/article/10.3390/biomedicines10020294/s1, Figure S1: Prism search flow; Figure S2: Gene identification search flow; Figure S3: All genes assessed by quantitative real-time quantitative PCR; Table S1: Table with relevant pathways for NAFLD progression; Table S2: Table with relevant previously identified genes and targets for NAFLD progression, Table S3: List of primers used in this study; Table S4: Dysregulated genes, annotated in Reactome; Table S5: Expression of previously identified molecular targets involved in the development and progression of chronic liver disease; Table S6: Complete data frame with all genes for all studies.
