# Supplementary material for: Impact of Maternal Obesity on Liver Disease in the Offspring: A Comprehensive Transcriptomic Analysis and Confirmation of Results in a Murine Model
Source: Biomedicines. 2022 Jan 27;10(2):294. doi: 10.3390/biomedicines10020294 (PMC8869223; doi:10.3390/biomedicines10020294)
Supplement: Supplementary file 1 [file biomedicines-10-00294-s001.zip › Index of Supplementary Files.pdf]

Index of supplementary files

**Supplementary figure S1:**	Prism search flow
**Supplementary figure S2:**	Gene identification search flow
**Supplementary figure S3:**	All genes assessed by quantitative real-time quantitative PCR
**Supplementary table S1:**	Table with relevant pathways for NAFLD progression
**Supplementary table S2:**	Table with relevant previously identified genes and targets for NAFLD progression
**Supplementary table S3:**	List of primers used in this study
**Supplementary table S4:**	Dysregulated genes, annotated in Reactome
**Supplementary table S5:**	Expression of previously identified molecular targets involved in chronic liver disease
**Supplementary table S6:**	Complete data frame with all genes for all studies
