# Supplementary material for: Impact of Maternal Obesity on Liver Disease in the Offspring: A Comprehensive Transcriptomic Analysis and Confirmation of Results in a Murine Model
Source: Biomedicines. 2022 Jan 27;10(2):294. doi: 10.3390/biomedicines10020294 (PMC8869223; doi:10.3390/biomedicines10020294)
Supplement: Supplementary file 1 [file biomedicines-10-00294-s001.zip › Supplementary Figures.pdf]

Supplementary figure S1: Prism search flow

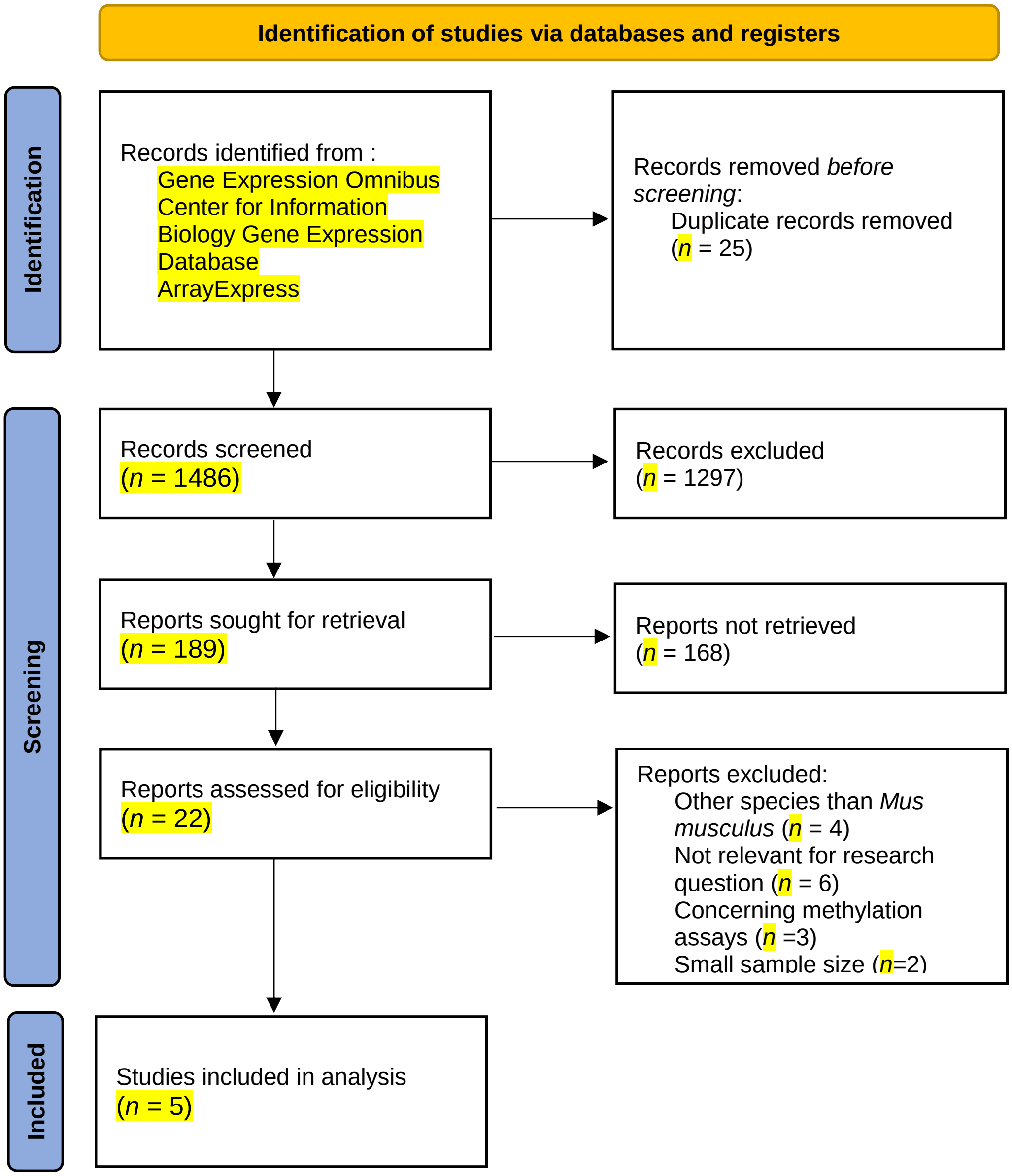

Supplementary figure S2: Gene identification search flow

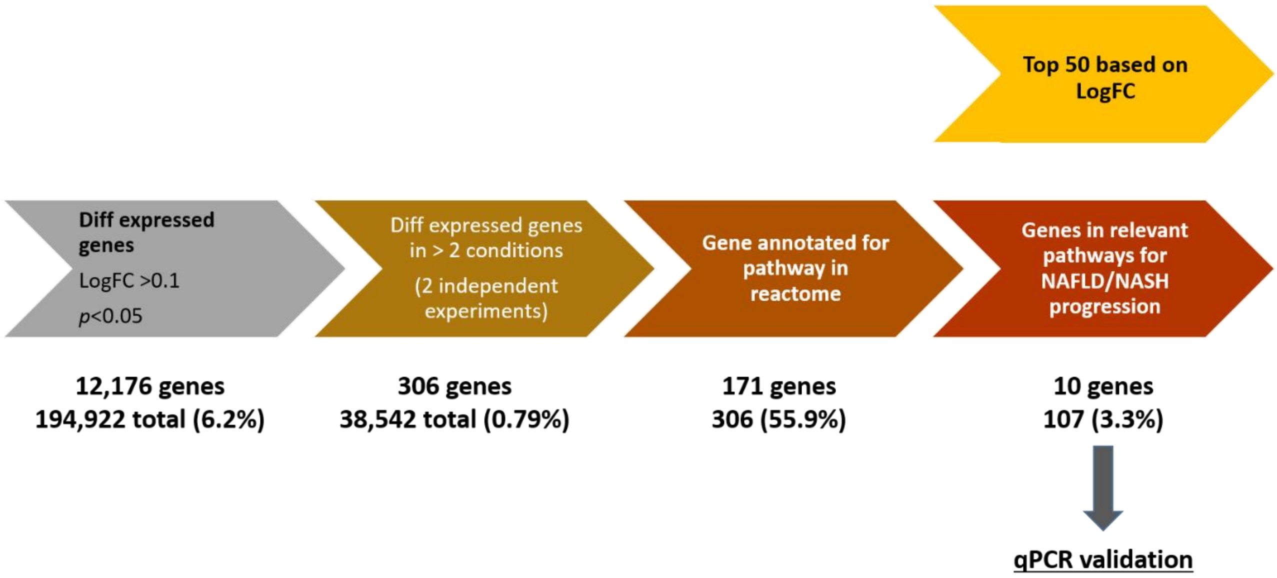

Figure S2. Illustration of the gene search flow to identify top dysregulated gene targets.

Supplementary figure S3: All genes assessed by quantitative real-time quantitative PCR

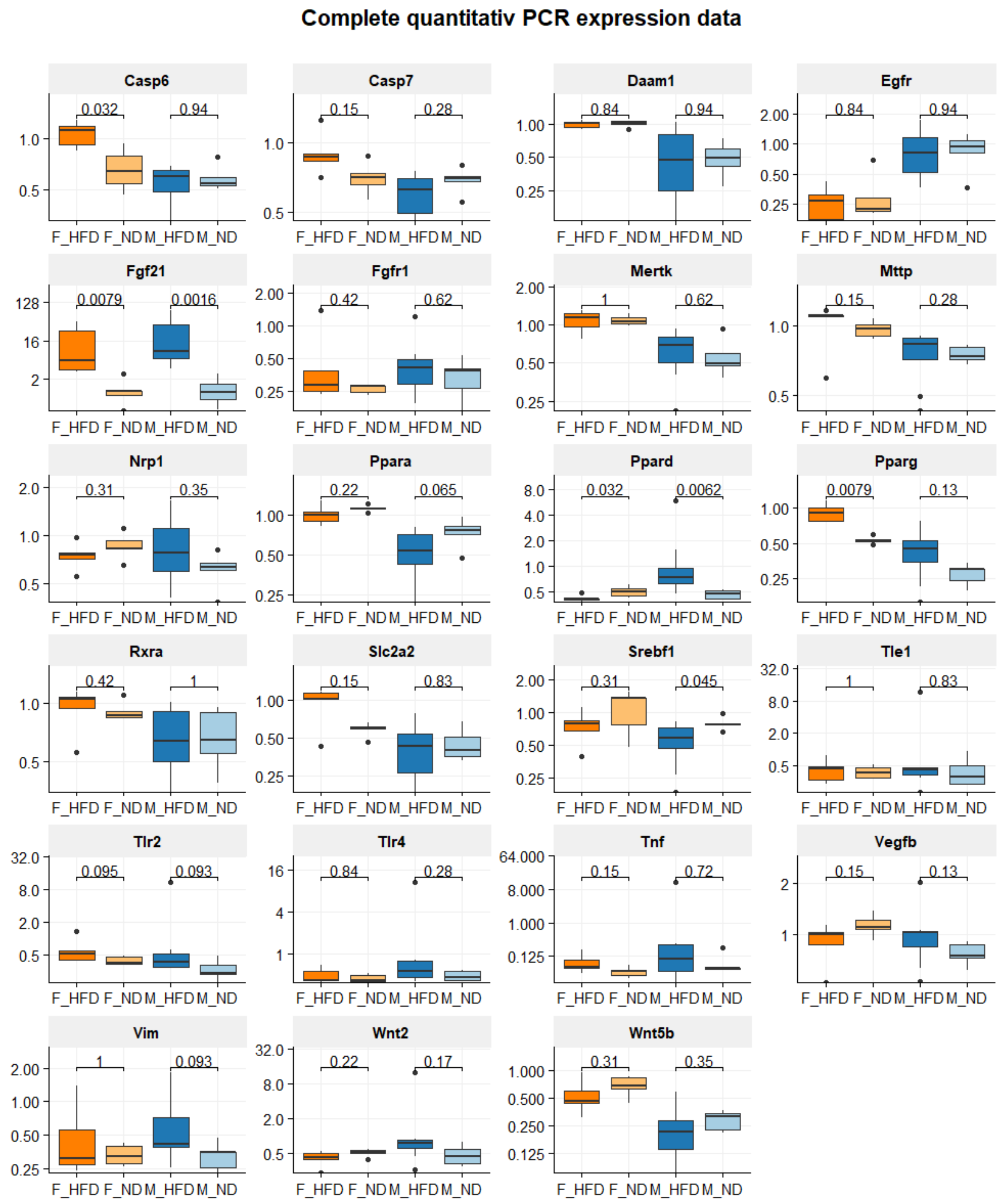

Figure S3. Maternal obesity dysregulates genes of the peroxisome proliferator activated receptor and caspase pathways in offspring. Quantitative real-time polymerase chain reaction gene expression results comparing the gene expression between offspring of obese and lean mothers. F_HFD : Female offspring born to obese mothers n=5, F_ND: Female offspring born to lean mothers n=5, M_HFD male offspring born to obese mothers n=8, M_ND male offspring born to lean mothers n=5. Statistical analysis: Wilcoxon signed rank test. Results are expressed as Median with interquartile ranges.
