# Supplementary material for: Impact of Maternal Obesity on Liver Disease in the Offspring: A Comprehensive Transcriptomic Analysis and Confirmation of Results in a Murine Model
Source: Biomedicines. 2022 Jan 27;10(2):294. doi: 10.3390/biomedicines10020294 (PMC8869223; doi:10.3390/biomedicines10020294)
Supplement: Supplementary file 1 [file biomedicines-10-00294-s001.zip › Supplementary Table S1_Pathways.pdf]

Supplementary Table S1

Pathway	Name	Description
PPAR	Peroxisome proliferator-activated receptors signaling pathway	Peroxisome proliferator-activated receptors (PPARs) are nuclear hormone receptors that are activated by fatty acids and their derivatives. PPARalpha plays a role in the clearance of circulating or cellular lipids via the regulation of gene expression involved in lipid metabolism in liver and skeletal muscle. PPARbeta/delta is involved in lipid oxidation and cell proliferation. PPARgamma promotes adipocyte differentiation
PI3K	PI3K-Akt signaling pathway	Intracellular signaling pathway important in regulating the cell cycle. It is directly related to cellular quiescence, proliferation, cancer, and longevity. PI3K activation phosphorylates and activates AKT, localizing it in the plasma membrane. AKT can have a number of downstream effects.
MAPK	Mitogen-activated protein kinase signaling pathway	Mitogen-activated protein kinase (MAPK) cascades are key signalling pathways that regulate a wide variety of cellular processes, including proliferation, differentiation, apoptosis and stress responses.
Wnt	Wnt signaling pathway	The Wnt signaling pathway is an ancient and evolutionarily conserved pathway that regulates crucial aspects of cell fate determination, cell migration, cell polarity, neural patterning and organogenesis during embryonic development.
VEGF	Vascular endothelial growth factor pathway	VEGF is the most prominent among the angiogenic cytokines and is believed to play a central role in the process of neovascularization, both in cancer as well as other inflammatory diseases.
Apoptosis	Apoptosis pathway	Apoptosis is a form of programmed cell death that occurs in multicellular organisms. Biochemical events lead to characteristic cell changes and death. These changes include blebbing, cell shrinkage, nuclear fragmentation, chromatin condensation, DNA fragmentation and mRNA decay.

TNF	Tumour-necrosis factor signaling pathway	The TNF signaling pathway plays an important role in various physiological and pathological processes, including cell proliferation, differentiation, apoptosis, and modulation of immune responses and induction of inflammation.
TLR	Toll-like receptor pathways	Toll-like receptors (TLRs) recognize distinct pathogen-associated molecular patterns and play a critical role in innate immune responses
