# Supplementary material for: Impact of Maternal Obesity on Liver Disease in the Offspring: A Comprehensive Transcriptomic Analysis and Confirmation of Results in a Murine Model
Source: Biomedicines. 2022 Jan 27;10(2):294. doi: 10.3390/biomedicines10020294 (PMC8869223; doi:10.3390/biomedicines10020294)
Supplement: Supplementary file 1 [file biomedicines-10-00294-s001.zip › Supplementary Table S2_Genes.pdf]

Supplementary Table S2

Symbol	Human analog	Aliases	Protein Name	Biological Process	Function
Adipoq	*ADIPOQ*		Adiponectin	Lipid metabolism	Metabolic and hormonal processes
Apoa4	*APOA4*		Apolipoprotein A4		Chylomicrons, VLDL
Casp6	*CASP6*		Caspase 6	Apoptosis	Cell apoptosis and programmed cell death
Ccl2	*CCL2*	*MCP1*	Monocyte Chemoattractant Protein 1		Disease with monocytic infiltration
Col13a1	*COL13A1*		Collagen alpha-1(XIII) chain		Regulates oxidative stress, inflammation and fibrogenesis
Cpt1a	*CPT1A*		Carnitine O-palmitoyltransferase 1	Lipid metabolism	Catalyzes the transfer of the acyl group of long-chain fatty acid-CoA conjugates onto carnitine
Fasn	*FASN*		Fatty acid synthase	Lipid metabolism	catalyzes the de novo biosynthesis of long-chain saturated fatty acids
Fgf19	*FGF19*		Fibroblast growth factor 19	Bile acid synthesis	Hormone that regulates bile acid synthesis
Fgf21	*FGF21*		Fibroblast growth factor 21	Carbohydrate metabolism	Important hepatokine with a multitude of metabolic functions
Fgfr1	*FGFR1*		Fibroblast growth factor receptor 1	Growth factor receptor binding	Tyrosine-protein kinase that acts as cell-surface receptor for fibroblast growth factors
Gckr	*GCKR*		Glucokinase regulatory protein	Carbohydrate metabolism	Regulates glucokinase by forming an inactive complex with this enzyme
Igfbp1	*IGFBP1*		Insuline like groth factor binding protein	Growth factor binding	Interacts with IGF, prolongs its halflife

Lep	*LEP*		Leptin	Energy homeostasis	Key player in the regulation of energy balance and body weight control
Lepr	*LEPR*		Leptin receptor	Energy homeostasis	Receptor for leptin
Mertk	*MERTK*		Tyrosine-protein kinase Mer	Innate immunity	Innate immunity, HSC activation
Mlxipl	*MLXIPL*	*ChREBP*	Carbohydrate-responsive element-binding protein	Energy homeostasis	Transcriptional repressor. Binds to the canonical and non-canonical E box sequences
Moxd1	*MOXD1*		DBH-like monooxygenase protein 1	Octopamine biosynthetic process	Catalyzes the conversion of dopamine to norepinephrine
Mtarc1	*MTARC1*		Mitochondrial amidoxime-reducing component 1	Nitrate metabolic process	Catalyzes the reduction of N-oxygenated molecules
Mttp	*MTTP*		Microsomal triglyceride transfer protein large subunit	Lipid metabolism	Required for the assembly and secretion of plasma lipoproteins
Nr1d1	*NR1D1*	*Rev-ErbA-Alpha*	Nuclear receptor subfamily 1 group D member 1	Biological rhytms	Transcriptional repressor, circadian Rhytm, various metabolic pathways
Nr1h4	*NR1H4*	*FXR, Fxr*	Bile acid receptor	Bile acid metabolism	Receptor for bile acids such as chenodeoxycholic acid, lithocholic acid and allocholic acid.
Pck1	*PCK1*		Phosphoenolpyruvate carboxykinase	Gluconeogenesis	Regulation of gluconeogenesis
Pnpla3	*PNPLA3*		1-acylglycerol-3-phosphate O-acyltransferase	Lipid metabolism	Potentiates profibrogenic and pro-inflammatory features of HSCs, increases hepatic lipid content
Ppara	*PPARA*		Peroxisome proliferator-activated receptor alpha	Transcription regulation	Key regulator of lipid metabolism. Regulates the peroxisomal beta-oxidation pathway of fatty acids.

Ppard	*PPARD*	*PPAR-Beta*	Peroxisome proliferator-activated receptor delta	Transcription regulation	Transcription factor, exercise mimetics, beta-catenin pathway
Pparg	*PPARG*		Peroxisome proliferator-activated receptor gamma	Transcription regulation	Nuclear receptor that binds peroxisome proliferators such as hypolipidemic drugs and fatty acids.
Ppargc1a	*PPARGC1A*	*PGC1a*	Peroxisome proliferator-activated receptor gamma coactivator 1-alpha	Transcription regulation	Transcriptional coactivator for steroid receptors and nuclear receptors. Greatly increases the transcriptional activity of PPARG.
Rxra	*RXRA*		Retinoic acid receptor	Transcription regulation	Receptor for retinoic acid that acts as a transcription factor
Saa1	*SAA1*		Serum amyloid A-1 protein	Acute phase	Major acute phase protein
Scd1	*SCD1*		Acyl-CoA desaturase 1	Lipid metabolism	Plays an important role in lipid biosynthesis
Serpina12	*SERPINA12*		Serine Protease inhibitor	Regulation of gluconeogenesis	Insulin resistance. Adipokine that modulates insulin action.
Sirt1	*SIRT1*		NAD-dependent protein deacetylase sirtuin-1	Biological rhytms and apoptosis	Involved in longevity and ageing associated cancer
Sirt3	*SIRT3*		NAD-dependent protein deacetylase sirtuin-3	Aerobic respiration	Important for regulating tissue-specific ATP levels
Slc2a2	*SLC2A2*	*GLUT2, Glut2*	Solute carrier family 2, facilitated glucose transporter member 2	Carbohydrate transport	Facilitative hexose transporter that mediates the transport of glucose and fructose
Socs2	*SOCS2*	*SSI2*	Suppressor of cytokine signaling 2	Growth regulation	Cytokine signaling, IGF signaling. Part of a classical negative feedback system that regulates cytokine signal transduction.
Sod2	*SOD2*		Superoxide dismutase	Oxygen homeostasis	Mitochondrial antioxidant

Srebf1	*SREBF1*	*SREBP-1c*	Sterol regulatory element-binding protein 1	Lipid metabolism	Key transcription factor that regulates expression of genes involved in cholesterol biosynthesis and lipid homeostasis
Tgfb1	*TGFB1*		Transforming growth factor beta-1 proprotein	Cell growth and differentiation	Multifunctional protein that regulates the growth and differentiation of various cell types.
Timp1	*TIMP1*		Tissue Inhibitor of Metalloproteinases 1	Negative regulation of catalytic process	Inhibitor of Metalloproteinases
Tlr2	*TLR2*		Toll-Like-Receptor 2	Innate immunity	Mediates the innate immune response to bacterial lipoproteins and other microbial cell wall components.
Tlr4	*TLR4*		Toll-Like-Receptor 4	Innate immunity	Mediates the innate immune response to bacterial lipopolysaccharide
Tnf	*TNF*		Tumor necrosis factor	Inflammatory response	It is mainly secreted by macrophages and can induce cell death and is a potent pyrogen
Ucp2	*UCP2*		Mitochondrial uncoupling protein 2	Adaptive thermogenesis	Uncouples oxidative phosphorylation from ATP synthesis. As a result, energy is dissipated in the form of heat
