# Supplementary material for: Impact of Maternal Obesity on Liver Disease in the Offspring: A Comprehensive Transcriptomic Analysis and Confirmation of Results in a Murine Model
Source: Biomedicines. 2022 Jan 27;10(2):294. doi: 10.3390/biomedicines10020294 (PMC8869223; doi:10.3390/biomedicines10020294)
Supplement: Supplementary file 1 [file biomedicines-10-00294-s001.zip › Supplementary Table S3_Primers.pdf]

Supplemental Table S3 Primers used in this study

Gene	NM accessions	Primer forward sequence	Primer reverse sequence
m-EGFR	NM_207655.2	GGACTGTGTCTCCTGCCAGAAT	TCCACAAACTCCCTTGGTTC
m-PPARg	NM_001127330.2	CTCACAATGCCATCAGGT	GCTGGTCGATATCACTGG
m-VEGFb	NM_011697.3	ACTGGGCAACACCAAGTCCGAA	CACATTGGCTGTGTTCTTCCAGG
m-WNT2	NM_023653.5	TCTGTCTATCTTGGGCATTCTG	TTCCTTCGCTATGTGATGTTTC
m-NRP1	NM_001358959.1	TGAGCCCTGTGGTCTATTCC	CGTCCTTCTTTCCAGTGGTC
m-VIM	NM_011701.4	CGGAAAGTGGAATCCTTGCAGG	AGCAGTGAGGTCAGGCTTGGAA
m-TLE1	NM_011599.5	GTGCCACCCTTGTGAGTTTT	GGTTTCTCAGGGGTAAGGATG
m-CASP7	XM_006526616.5	CCGTCCACAATGACTGCTCTTG	CCCGTAAATCAGGTCCTCTTCC
m-WNT5b	NM_001271758.1	TGTCAGTTGTATCAGGAGCACA	GTGAAGGCAGTCTCTCGGCTA
m-DAAM1	NM_026102.3	GAACACAAGCATGAGCTGGA	AACACCTCCTCAGAGCCAGA
m-TLR2	NM_011905.3	ACAGCAAGGTCTTCCTGGTTCC	GCTCCCTTACAGGCTGAGTTCT
m-FGF21	NM_020013.4	CTGGGGGTCTACCAAGCATA	GCCTACCACTGTTCCATCCT
m-FGFR1	NM_010206.3	ACCAAGAAGAGCGACTTCCA	AACCAGGAGAACCCCAGAGT
m-RXRA	NM_011305.3	GTGAAAGATGGGATTCTCCTGGC	GTCACGCATCTTAGACACCAGC
m-CASP6	NM_009811	CATGGTGGATCACCAGACAGAC	GGAGCCATTCACAGTTTCTCGG
m-TLR4	NM_021297.2	GGCAGCAGGTGGAATTGTAT	AGGATTCGAGGCTTTTCCAT
m-PPARa	NM_011144.6	ACGATGCTGTCCTCCTTGATG	GTGTGATAAAGCCATTGCCGT
m-PPARd	NM_011145.3	TCCCAGGGTCAGAAGCTAGA	CCAGTCTGGATGCTGCTACA
m-TNF	NM_013693.3	CTGGGAGTAGACAAGGTACAACCC	CACCAAGTGGAGGAGCAGCT
m-SLC2A2	NM_031197.2	AAGGATCTGCTCACATAGTCACT	TTGCAGCCAACATTGCTTTGA
m-MTTP	NM_001163457.2	CCAGGAAAGGTTCCTCTATGCC	GACTCTCTGATGTCGTTGCTTGC
m-MERTK	NM_008587.2	ATCATCCTCGGCTGCTTCTGTG	ACGACCAGTTGGGAATCCTCCT
m-SREBF1	NM_011480.4	GCAATCCTTTGGCCTCGCTTTT	CAGCAACCACTGGGTCCAATTAGA
