# Supplementary material for: Impact of Maternal Obesity on Liver Disease in the Offspring: A Comprehensive Transcriptomic Analysis and Confirmation of Results in a Murine Model
Source: Biomedicines. 2022 Jan 27;10(2):294. doi: 10.3390/biomedicines10020294 (PMC8869223; doi:10.3390/biomedicines10020294)
Supplement: Supplementary file 1 [file biomedicines-10-00294-s001.zip › Supplementary Table S5_ExpressionGenesOfInterest.pdf]

Supplemental Table S4: Expression of previously identified molecular targets involved in the development and progression of chronic liver disease

Gene	Gene name	Function	Main Associated pathways	Nb Comparison up	Nb Comparison down	Median logFC	Fisher's *p*-value
Pparg	Peroxisome proliferator-activated receptor gamma	Nuclear receptor involved in critical metabolism regulations of various cell types, notably adipocyte differentiation.	• MAPK-Erk	0	3	0.268	1.8×10^{-3}
Tlr2	Toll-like receptor 2	Membrane surface receptor essential for pathogen recognition and innate immune response activation.	• NF-kappa-B<br>• MAPK-Erk	0	2	0.135	3.3×10^{-2}
Fgf21	Fibroblast growth factor 21	Hepatokine involved in mitogenic activities. Major regulator of energy homeostasis.	• MAPK-Erk<br>• Insulin signaling	2	0	0.131	0.079
Fgfr1	Fibroblast growth factor receptor 1	Receptor tyrosine kinase that plays a fundamental role in embryogenesis and cell development.	• MAPK-Erk<br>• PI3K/AKT	0	1	0.064	0.092
Rxra	Retinoid X receptor alpha	Transcriptional regulator protein involved in carcinogenesis and mineral and lipid metabolism.	• Calcium signaling<br>• MAPK-Erk<br>• PI3K/AKT	1	1	0.061	0.127
Casp6	Caspase-6	Apoptosis-related cysteinyl aspartate proteinase. Protein coding gene for cell death execution via activation cascades.	• Apoptosis	0	0	0.111	0.080
Tlr4	Toll-like receptor 4	Membrane surface receptor essential for pathogen recognition and innate immune response activation.	• NF-kappa-B<br>• MAPK-Erk	0	0	0.061	0.607
Ppara	Peroxisome proliferator-activated receptor alpha	Nuclear receptor regulating lipid metabolism and homeostasis in the liver. Important factor in fatty liver disease and carcinogenesis.	• MAPK-Erk	0	0	0.053	0.667
Ppard	Peroxisome proliferator-activated receptor delta	Nuclear receptor participating in energy metabolism regulations. Synergizes with RXR family.	• MAPK-Erk	0	0	0.046	0.819
Tnf	Tumor necrosis factor	Key cytokine of the inflammatory cascade and cell death regulation. Implicated in various autoimmune and metabolic diseases.	• NF-kappa-B<br>• MAPK-Erk	0	0	0.039	0.733

Abbreviations: Nb comparison up: Number of Gene Enrichments sets in which the gene is upregulated; Nb comparison down: Number of Gene Enrichments sets in which the gene is downregulated; LogFC: log2 Fold Change
